# The Desmoplastic Stroma of Pancreatic Cancer: Multilayered Levels of Heterogeneity, Clinical Significance, and Therapeutic Opportunities

**DOI:** 10.3390/cancers14133293

**Published:** 2022-07-05

**Authors:** Yohei Masugi

**Affiliations:** 1 Division of Diagnostic Pathology, Keio University School of Medicine, Tokyo 1608582, Japan; masugi@z6.keio.jp; Tel.: +81-3-5363-3764; Fax: +81-3-3353-3290; 2 Department of Pathology, Keio University School of Medicine, Tokyo 1608582, Japan

**Keywords:** pancreatic ductal adenocarcinoma, desmoplasia, fibroblast, collagen, immunotherapy

## Abstract

**Simple Summary:**

Pancreatic cancer is a highly malignant disease with treatment resistance to standardized chemotherapies. In addition, only a small fraction of patients with pancreatic cancer has, to date, actionable genetic aberrations, leading to a narrow therapeutic window for molecularly targeted therapies or immunotherapies. A lot of preclinical and translational studies are ongoing to discover potential vulnerabilities to treat pancreatic cancer. Histologically, human pancreatic cancer is characterized by abundant cancer-associated fibrotic stroma, called “desmoplastic stroma”. Recent technological advances have revealed that desmoplastic stroma in pancreatic cancer is much more complicated than previously thought, playing pleiotropic roles in manipulating tumor cell fate and anti-tumor immunity. Moreover, real-world specimen-based analyses of pancreatic cancer stroma have also uncovered spatial heterogeneity and an intertumoral variety associated with molecular alterations, clinicopathological factors, and patient outcomes. This review describes an overview of the current efforts in the field of pancreatic cancer stromal biology and discusses treatment opportunities of stroma-modifying therapies against this hard-to-treat cancer.

**Abstract:**

Pancreatic cancer remains one of the most lethal malignancies and is becoming a dramatically increasing cause of cancer-related mortality worldwide. Abundant desmoplastic stroma is a histological hallmark of pancreatic ductal adenocarcinoma. Emerging evidence suggests a promising therapeutic effect of several stroma-modifying therapies that target desmoplastic stromal elements in the pancreatic cancer microenvironment. The evidence also unveils multifaceted roles of cancer-associated fibroblasts (CAFs) in manipulating pancreatic cancer progression, immunity, and chemotherapeutic response. Current state-of-the-art technologies, including single-cell transcriptomics and multiplexed tissue imaging techniques, have provided a more profound knowledge of CAF heterogeneity in real-world specimens from pancreatic cancer patients, as well as in genetically engineered mouse models. In this review, we describe recent advances in the understanding of the molecular pathology bases of pancreatic cancer desmoplastic stroma at multilayered levels of heterogeneity, namely, (1) variations in cellular and non-cellular members, including CAF subtypes and extracellular matrix (ECM) proteins; (2) geographical heterogeneity in relation to cell–cell interactions and signaling pathways at niche levels and spatial heterogeneity at locoregional levels or organ levels; and (3) intertumoral stromal heterogeneity at individual levels. This review further discusses the clinicopathological significance of desmoplastic stroma and the potential opportunities for stroma-targeted therapies against this lethal malignancy.

## 1. Introduction

Pancreatic cancer remains the most lethal malignancy, with a 5-year survival rate of approximately 10%, even in developed countries [1]. As the worldwide incidence rapidly increases, pancreatic cancer is becoming a dramatically increasing cause of cancer mortality [2,3]. In the United States, pancreatic cancer is projected to become the second most common cause of cancer death by approximately 2026 [4]. At the time of diagnosis, most pancreatic cancer patients have no symptoms but have metastatic and/or locally advanced disease, making it difficult to undergo curative surgery. Even after successful pancreatectomy and undergoing adjuvant therapies, many patients with PDAC experience tumor recurrence due to treatment resistance to standard chemotherapies.

Since more than 90% of adult pancreatic malignancies are pancreatic ductal adenocarcinoma (PDAC), ‘pancreatic cancer’ is colloquially used as a synonym for PDAC. PDAC is an invasive pancreatic epithelial tumor with tubule-like structures resembling pancreatic ducts. PDAC develops through the accumulation of genetic and epigenetic aberrations, including four common driver genes: *KRAS*, *TP53*, *CDKN2A*, and *SMAD4* [5,6,7,8,9,10,11,12,13]. Recently, cancer genome sequencing efforts have uncovered a number of genomic alterations involved in pancreatic carcinogenesis [14,15,16,17,18,19,20]. However, to date, a limited fraction of individuals with pancreatic cancer appear to have actionable genetic alterations, including *BRCA1* and *BRCA2* mutations, *neurotrophic receptor tyrosine kinase* (*NTRK)* gene fusions, and microsatellite instability (MSI)-high [11,17,21,22,23,24,25,26]. In addition, the single use of immune checkpoint inhibitors (ICIs) targeting programmed cell death 1 (PDCD1), CD274 (also known as programmed cell death 1 ligand 1, PD-L1, or B7-H1), and cytotoxic T lymphocyte-associated protein 4 (CTLA4) has shown no effectiveness in metastatic pancreatic cancers [27]. Therefore, many preclinical and translational studies are ongoing to discover potential vulnerabilities and therapeutic opportunities to treat PDAC [28,29,30].

The histology of human PDAC has been unique. Compared to adenocarcinomas in other organs, PDAC generally has a prominent fibrotic stroma, called “desmoplastic stroma”. The fraction of carcinoma cells within the tumor bed is often less than 20% in patients with PDAC [31], which likely hinders biological and molecular investigations of pancreatic cancer pathogenesis. The desmoplastic stroma is comprised of heterogeneous cellular and non-cellular members, such as fibroblasts, immune cells, blood vessels, lymphatic vessels, and extracellular matrix (ECM) proteins [32]. Numerous previous studies have revealed that these stromal elements can regulate the tumor molecular mechanisms underlying various cellular functions, including proliferation, survival, senescence, apoptosis, cell motility, invasion, and metastasis [32,33,34,35,36,37]. Furthermore, emerging evidence derived from single-cell transcriptome analyses has underscored the importance of fibroblastic subpopulations with different functional and phenotypic characteristics [38,39]. Additionally, tumor cell fate (and, eventually, tumor aggressiveness) is primarily dictated by the dynamic interactions between carcinoma cells and the surrounding stromal factors and/or architectures, attesting to the need for a deeper understanding of geographical heterogeneity in desmoplastic stroma within PDAC tissues [40,41,42,43]. Recent analyses of human real-world data on PDAC have revealed the clinical significance of intertumoral stromal heterogeneity [44], likely providing new insights into treatment opportunities and harnessing stromal therapies to target potential vulnerabilities of this deadly cancer.

**Figure 1:**
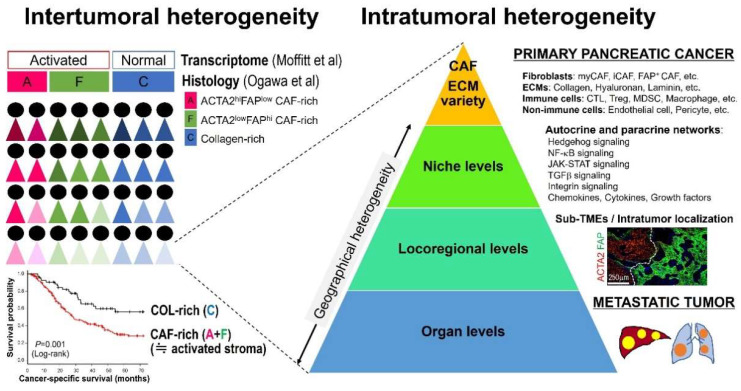
Multilayered levels of pancreatic ductal adenocarcinoma (PDAC) stromal heterogeneity. PDAC desmoplastic stroma is highly heterogenous between patients and even in a tumor at multilayered levels. Abbreviations: CAF, cancer-associated fibroblast; CTL, cytotoxic T cell; ECM, extracellular matrix; iCAF, inflammatory cancer-associated fibroblast; MDSC, myeloid-derived suppressor cell; myCAF, myofibroblast cancer-associated fibroblast; TME, tumor microenvironment; Treg, regulatory T cell.

An increasing number of papers have emphasized “heterogeneity” to describe the complexity of the tumor microenvironment (TME) in various types of tumors, including PDAC. However, there is no consensus over a definition of the “heterogeneity” concept, despite widespread use of this term. For example, when referring to stromal heterogeneity, some have in mind only variations in stromal elements, while others may include spatial distributions and population-level differences. Numerous scientific findings stemming from distinct research fields are obviously essential for establishing the “stromal heterogeneity” field, but different studies often use the term “heterogeneity” differently. Moreover, we should acknowledge that different levels of “stromal heterogeneity” have differing clinical relevance. For instance, identification of a specific cell subtype within intratumoral elements would promote drug development against the specific target, while the notion of intertumoral heterogeneity would attest to the importance of patient stratification and biomarker discovery for precision medicine approaches. Hence, this review summarizes the current knowledge of PDAC stromal heterogeneity, distinguishing three distinct layers: (1) non-cellular and cellular variations, (2) geographical heterogeneity and cell–cell interactions, and (3) intertumoral heterogeneity (Figure 1). We also discuss the clinicopathological significance of the stromal heterogeneity of PDAC and further introduce several clinical trials evaluating agents targeting stromal elements to discuss therapeutic opportunities for stroma-modifying therapies.

## 2. Extracellular Matrix

The ECM is an extracellular meshwork containing structural proteins, proteoglycans, adaptor proteins, and tissue remodeling enzymes. A variety of ECM proteins and macromolecules, derived either from stromal cells or tumor cells, are deposited in the desmoplastic stroma of PDAC [45]. Compared to the healthy pancreas, the ECM in the tumor is characterized by markedly increased stiffness and density [46]. Tumor ECM not only serves as a simple structural support but also plays a pivotal role in modifying tumor cell behavior. For example, aberrant ECM accumulation attributes to increased interstitial pressure and the collapse of capillary vessels, leading to the decreased delivery of blood flow and therapeutic agents, which results in a hypoxic microenvironment and altered drug response [47,48,49]. Signaling pathways, such as the integrin signaling pathway, also contribute to the regulation of cellular machinery in pancreatic cancer cells [50]. In addition, many growth factors stored in the ECM can enhance tumor cell proliferation, survival, and cell motility [51]. Matrix stiffness itself has promoted the epithelial−mesenchymal transition (EMT) [52]. On the other hand, like different types of neoplasms, rigid fibrous tissues may function as physical barriers to restrain tumor growth by capsulizing pancreatic cancer nests under certain circumstances.

In this section, we reviewed the heterogeneous roles of major non-cellular stromal constituents of the pancreatic cancer microenvironment and discussed the potential opportunities of ECM-targeting therapies.

### 2.1. Collagens

More than 90% of ECM proteins are produced by stromal cells such as fibroblasts [45]. Among the stromal cell-derived ECM molecules, collagens are the predominant components, accounting for more than 90% of ECM proteins in pancreatic cancer stroma and chronic pancreatitis [45]. Collagens include 29 known superfamily members in vertebrates [49]. Proteomic analyses have revealed that approximately 90% of the total collagens in PDAC tissues are type I and type III fibrillar-type collagens [45], which are predominantly produced by stromal cells, especially ACTA2, the HUGO (Human Genome Organization)-approved official symbol for actin alpha 2, smooth muscle; HGNC ID: 130; it is also known as alpha smooth muscle actin or αSMA-expressing fibroblasts [53].

Several anti-fibrotic approaches are ongoing in the treatment of PDACs (Table 1). One such strategy includes the inhibition of the lysyl oxidase (LOX) family proteins, which are essential enzymes that facilitate the cross-linking of collagen molecules, leading to the stabilization and integrity of collagen fibrils [54]. Many reports have suggested that the upregulation of LOX and lysyl oxidase like 2 (LOXL2) is associated with reduced survival in many malignancies, although there are conflicting data on the clinical significance of LOX family enzymes [55,56]. A recent study has shown that LOXL2 promotes EMT and stemness and increases the metastatic capacity of murine PDACs [57]. However, a phase II clinical trial has demonstrated that an anti-LOXL2 antibody simtuzumab, in combination with gemcitabine, failed to show any benefit in treating patients with metastatic PDAC [58]. Another approach involves the use of renin−angiotensin-inhibiting hypotensive agents, anticipating their anti-fibrotic effect. Indeed, an angiotensin receptor antagonist, losartan, has shown encouraging results when combined with FOLFIRINOX (which is made up of 5-Fluorouracil, Oxaliplatin, Irinotecan, and Leucovorin) as a neoadjuvant therapy against locally advanced PDACs in a phase II study [59].

Collagens are major integrin ligands that mediate intercellular signaling, including focal adhesion kinases (FAKs) [50]. The FAK is a non-receptor-type tyrosine kinase which has been hyperactivated in many tumor types to regulate multiple cellular functions of tumor cells, including cell−matrix adhesion, proliferation, apoptosis, migration, and invasion. A previous study has shown that FAK inhibition in murine PDAC models has enhanced the anti-tumor effect of checkpoint immunotherapies [66]. In addition, FAK inhibition has increased CD8^+^ T cell infiltrates and reduced the number of fibroblast activation protein α (FAP)-expressing fibroblasts, myeloid-derived suppressor cells (MDSCs), macrophages, and regulatory T cells (Tregs) within mice tumor tissues, suggesting the promising role of FAK pathway inhibition in reprogramming PDAC stroma to abrogate immunotherapy resistance [66]. A phase I/IIa clinical trial is ongoing to test the effect of a FAK inhibitor, defactinib, in combination with gemcitabine and pembrolizumab in pancreatic cancers. Preliminary data from this trial have reported decreased FAK activity, as well as reduced intratumoral T cell infiltration, in paired biopsy specimens from the patients [60].

### 2.2. Hyaluronan

The hyaluronan (hyaluronic acid) is a large glycosaminoglycan, distributed across the whole body [67]. Half of the total hyaluronan in the body are present in the cutaneous tissue, holding water to plump and moisturize the skin [68]. Indeed, 1 g of hyaluronan can absorb nearly 6 kg of water molecules [69]. Hyaluronan is abundantly accumulated in the tumor stroma of various malignancies, including PDAC. Tumor stromal hyaluronan is primarily produced by activated fibroblasts in the TME [49]. Hyaluronan is the primary ligand of the cell surface receptor CD44, which can regulate cell proliferation, stemness, motility, and metastasis during tumor evolution and progression [67,70]. The aberrant intratumoral deposition of hyaluronan leads to a remarkable increase in interstitial fluid pressure, hindering microcirculation and immune cell infiltrates via the collapse of small vessels [71,72]. In the KPC genetically engineered mouse model (GEMM), the interstitial fluid pressure in the tumor is approximately ten times greater than that in a healthy pancreas [73]. After a single intravenous dose of a chemically modified hyaluronidase pegylated recombinant human hyaluronidase 20 (PEGPH20), intratumoral interstitial fluid pressure in mice PDAC models was decreased within 2 h to approach the range for a normal pancreas 24 h after treatment [73]. Moreover, KPC GEMMs treated with gemcitabine with or without PEGPH20 have shown the benefit of PEGPH20 in terms of a reduced vascular collapse and enhanced intratumoral delivery of gemcitabine agents, leading to lower metastatic incidence and prolonged PDAC survival [73,74].

In accordance with the promising effects of hyaluronan degradation in enhancing therapeutic response in preclinical models of PDAC, PEGPH20 has undergone several clinical trials. A randomized phase II trial investigating the potential benefit of PEGPH20 in combination with the standard gemcitabine plus nab-paclitaxel chemotherapy has shown a better progression-free survival in the investigation arm in metastatic pancreatic cancer patients (HR, 0.51; 95% CI, 0.26–1.00) [61]. However, another phase Ib/II clinical trial in 138 treatment naive PDACs with metastatic disease demonstrated a detrimental effect of PEGPH20 when combined with modified FOLFIRINOX (median overall survival, 7.7 months for the modified FOLFIRINOX (mFOLFIRINOX) plus PEGPH20 group vs. 14.4 months for the mFOLFIRINOX alone group) [62]. It is noteworthy that this unexpected result was likely explained by increased toxicity, which led to a significantly shortened treatment duration in nearly half of the patients treated with the PEGPH20-containing regimen [62]. Intriguingly, four out of 55 metastatic PDAC patients in the mFOLFIRINOX plus PEGPH20 group showed a complete response [62]. A recent phase III randomized controlled trial comparing gemcitabine plus nab-paclitaxel with or without PEGPH20 in the treatment of metastatic PDACs was unfortunately terminated, with adverse study outcomes (i.e., median overall survival, 11.2 months for the chemotherapy plus PEGPH20 group vs. 11.5 months for the chemotherapy alone group) [63]. The trials’ failure has discouraged the attempt to harness a hyaluronan-degrading PEGPH20 agent to sensitize the conventional chemotherapies against PDACs.

### 2.3. Laminins

Specific laminins within the PDAC stroma have been derived from cancer cells [45]. The laminin α3β3γ2 (known as laminin 5), a triplex protein made of laminin subunit alpha 3 (LAMA3), laminin subunit beta 3 (LAMB3), and laminin subunit gamma 2 (LAMC2), are the significant laminins accumulated in PDAC stroma [75]. These laminin molecules and the type IV collagens, other upregulated non-fibrillar collagens in PDAC stroma, independently self-assemble to form the basement membrane in the soft stromal matrix [49]. The laminin α3β3γ2 is a primary ligand of integrin α6β4, a heterodimer of integrin subunits, integrin subunit alpha 6 (ITGA6) and integrin subunit beta 4 (ITGB4), to activate intratumoral integrin-dependent signaling. ITGB4 overexpression has been associated with shortened survival in various cancers, including PDACs [76]. Co-overexpressed ITGB4 and LAMC2 proteins likely contribute to the formation of hemidesmosomes to provide matrix anchorage at the invasive front of human PDAC tissues [76,77]. Moreover, upregulations of ITGB4 and LAMC2 in tumor cells induces EMT properties that promote cancer invasiveness, therapeutic resistance, and metastatic capacity [76,78,79,80]. Tumor ITGB4 appears to play a role in the regulation of stem cell properties and immunosuppression [81,82]. Future translational and clinical studies are warranted to investigate therapeutic opportunities to target the α6β4 integrin–laminin α3β3γ2 axis in the treatment of pancreatic cancer [83,84].

## 3. Fibroblast Heterogeneity

Fibroblasts are mesenchymal lineage cells that exist in the stroma of nearly all organs. Fibroblasts are the primary source of ECM proteins, including collagens and hyaluronans, to support and maintain tissue architectures [49]. Fibroblasts in the tumor tissues are often called cancer-associated fibroblasts (CAFs). There are considerable differences in the phenotypes and cellular functions of CAFs in tumor tissues and fibroblasts in healthy organs [85]. CAFs play a key role in producing matrix-reconstructing enzymes and secreting various growth factors, cytokines, and exosomes in the tumor microenvironment, thereby regulating tumor growth, invasion, metastasis, and therapeutic resistance [86,87,88]. Recent technologies have shown that fibroblasts in PDAC tissues are, phenotypically and functionally, much more heterogeneous than previously thought. This section reviews the cellular heterogeneity of fibroblasts and discusses the significance of different CAF subpopulations in the tumor biology of PDAC.

### 3.1. CAF Origins

Tissue-resident fibroblasts in the normal pancreas are pancreatic stellate cells (PSCs). PSCs primarily localize just around the pancreatic acini and ducts [89]. PSCs produce collagens, laminins, fibronectins, and ECM-remodeling enzymes (i.e., MMPs and tissue inhibitors of MMPs (TIMPs)) at a physiological state to support normal pancreatic parenchyma and maintain homeostasis of exocrine and endocrine functions [86]. More importantly, PSCs play a primary role in monitoring the leakage of pancreatic juice that contains potent autolytic enzymes secreted by acinar cells, quickly responding to various types of injuries in pathologic states, such as acute pancreatitis. Quiescent PSCs are characterized by the presence of vitamin A-storing cytoplasmic lipid droplets [90]. Once activated, PSCs reduce intracellular lipid droplets and simultaneously express activation markers (i.e., ACTA2 and FAP) and upregulate ECM-related molecules, exhibiting similar phenotypes to CAFs. A similarity can be observed between the CAFs in PDAC tissues and the activated PSCs in PDAC precursors, including pancreatic intraepithelial neoplasia (PanIN) and intraductal papillary mucinous neoplasia (IPMN) [91,92,93,94,95,96,97,98]. Studies suggest that this preinvasive lesion-associated PSC activation results from complex stimuli: (1) the latent obstruction of pancreatic ducts and (2) paracrine factors secreted from neighboring neoplastic cells [77]. A recent study using a lineage-tracing platform has revealed differential contributions between two differentially located resident fibroblasts (GLI family zinc finger 1 (GLI1)+ fibroblasts and homeobox B6 (HOXB6)+ fibroblasts) to CAF generation during murine PDAC carcinogenesis [99].

PSCs have long been considered a principal source of CAFs in PDACs. However, previous studies in various tumor types have indicated multiple cell origins for CAFs, including resident fibroblasts, bone marrow-derived mesenchymal stem cells, adipose-derived stem cells, mesothelial cells, endothelial cells, and epithelial cells [90,100,101,102,103,104]. A recent study has suggested a pro-tumorigenic contribution of PSC-derived CAFs, although PSC-derived CAFs are a minor subset of the total CAFs present in PDAC tissues [105]. Taken together, fibroblasts from multiple origins may contribute to a wide range of heterogeneity in CAF subtypes within pancreatic cancer tissues. Future studies may clarify how CAFs from different cellular origins influence tumor biology in patients with pancreatic ductal adenocarcinomas.

### 3.2. CAF Markers

The lack of sensitive and specific protein markers for fibroblast lineage cells has long been a limitation to the comprehensive understanding of the CAFs’ biology. For example, desmin (DES) or glial fibrillary acidic proteins (GFAP) are PSC markers, but they are not specific to fibroblasts; DES and GFAP are more abundantly expressed in muscle lineage cells and glial cells, respectively. Much effort has been devoted to exploring CAF-specific markers. ACTA2 is a famous histological marker of smooth muscle cells and myofibroblasts in the clinical practice of diagnostic pathology. Due to the similarities between CAFs and activated PSCs, ACTA2 has long been a gold standard marker for detecting CAFs. Although a large proportion of fibroblasts in pancreatic cancer tissues appear to express ACTA2, not all fibroblasts are detectable by an anti-ACTA2 antibody. Reports suggest that ACTA2 and FAP may characterize somewhat overlapping but distinct CAF subpopulations in several tumor types [44,91,106]. Other fibroblast markers, including S100A4 (also known as fibroblast-specific protein-1 or FSP1), platelet-derived growth factor receptor (PDGFR), and podoplanin (PDPN), appear to define different CAF subsets [77,107,108]. Considering the broad spectrum of CAFs, the use of a multiplex panel of fibroblast markers, not a single marker, likely plays a major role in describing the total CAF populations and multiple distinct CAF subtypes [44].

### 3.3. CAF Subtypes

The myofibroblast, an activated form of fibroblast, is a fibroblast subpopulation traditionally defined using morphological methods. Myofibroblasts were distinguished from standard fibroblasts by the presence of actin filaments under electron microscopy. Then, the biochemical and immunohistochemical characteristics of myofibroblasts were further clarified. Myofibroblasts may play a key role in tension development in many pathological states, such as wound healing [109]. Myofibroblasts are additionally characterized by ACTA2 expression at a light microscopic level. Studies suggest that CAFs and myofibroblasts share many phenotypic and functional traits [28].

**Figure 2:**
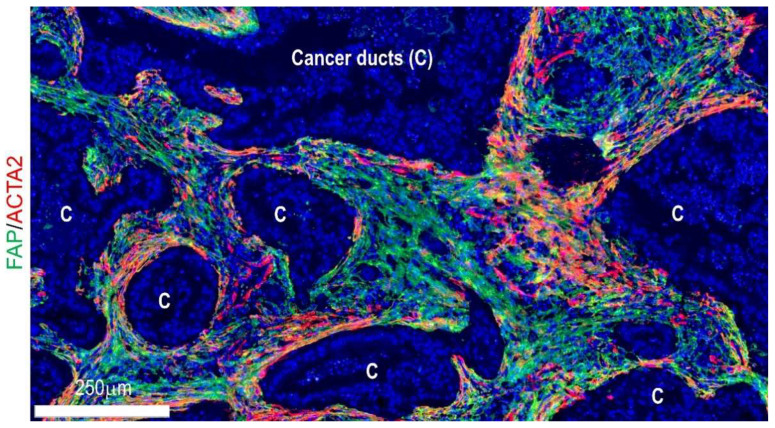
Visualization of fibroblast subpopulations in human pancreatic cancer tissues using fluorescent immunohistochemistry for ACTA2 and FAP.

Recently, findings in co-cultures of PSCs and PDAC organoids from murine models have identified a novel CAF subpopulation with different transcriptome profiles from myofibroblasts [40]. When directly co-cultured with tumor organoids, PSCs showed an elevated level of ACTA2 and myofibroblast-like phenotypes [40]. In contrast, when indirectly co-cultured using trans-wells, PSCs did not exhibit ACTA2 upregulation but expressed various inflammatory cytokines, including interleukin 6 (IL6), C-X-C motif chemokine ligand 12 (CXCL12), and LIF interleukin 6 family cytokine (LIF); these were termed inflammatory CAFs (iCAFs) [40]. Consistent with these experiments, ACTA2-expression was more frequent in juxta-tumoral fibroblasts within human and murine PDAC tissues, while IL6- or FAP-expressing fibroblasts were noted in fibroblasts that were more distal from carcinoma ducts (Figure 2) [40,44]. These lines of evidence indicate that the fibroblast’s fate decision can be dictated in a context-dependent manner (further discussed in Section 4.1). Thereafter, single-cell transcriptome analyses confirmed the presence of myofibroblast CAFs (myCAFs) and iCAFs as distinct CAF subtypes in human PDACs [38]. In addition, many study groups proposed other CAF subtypes with distinct transcriptome profiles and presumably differing functions, for example, antigen-presenting CAFs (apCAFs), desmoplastic CAFs (dCAFs), proliferating CAFs, complement-secreting CAFs (csCAFs) etc. [38,110,111,112].

## 4. Geographical Heterogeneity

During pancreatic carcinogenesis, tumor cells interact with fibroblasts to shape a unique stroma favor, supporting tumor growth, invasion, and immunosuppression [53,113,114,115,116,117,118,119,120]. Research is underway to understand the complicated signaling networks between tumor cells, fibroblast subtypes, and immune cells to develop stroma-modifying therapies against PDACs [121,122,123,124,125]. Moreover, spatial analyses have revealed significant differences in the tumor−immune microenvironment using different intratumoral locations and organ sites [126,127]. This section discusses the geographical/spatial heterogeneity of PDAC stroma at niche, locoregional, and organ levels.

### 4.1. Cell–Cell Interactions and Signaling at Niche Levels

Since the direction of CAF phenotypes appears to largely depend on the passive diffusion of paracrine and/or autocrine factors [41,123,128,129], spatial arrangement and cell–cell distances within a localized “niche” (presumably 100 μm) could be substantial. For example, indirect co-culture with KPC mice-derived PDAC cells or treatment with tumor-conditioned media promoted iCAF differentiation from PSCs with the rapid phosphorylation of the RELA proto-oncogene, NF-kB subunit (RELA), whereas the treatment of an NF-κB inhibitor attenuated the iCAF-associated gene expression phenotypes [41]. In addition, tumor-derived interleukin 1 alpha (IL1A) induces LIF upregulation in PSCs, leading to the activation of JAK-STAT signaling pathways to promote iCAF induction [41]. Evidence also suggests a crucial role of the LIF-mediated phosphorylation of the signal transducer and activator of transcription 3 (STAT3) in maintaining the iCAF phenotype [41]. These findings attest to the importance of tumor-derived humoral factors in initiating and maintaining iCAFs in the PDAC tumor microenvironment. Moreover, studies have suggested that several CAF-derived cytokines, including IL6 and LIF, can induce an EMT phenotype in tumor cells and an immune evasion in the tumor microenvironment to promote tumor progression in murine PDAC models [123,128,130].

A histological investigation into human PDAC tissues has demonstrated that fibroblasts tightly surrounding well-differentiated cancer ducts frequently show ACTA2 overexpression within the collagen-rich stroma [44], suggesting that spatial cues likely direct myofibroblast phenotypes as well as tumor cell morphology. Transforming growth factor beta 1 (TGFB1) has been a major driver of the morphological and functional phenotypes of myofibroblasts [131,132]. Previous reports have revealed that ACTA2-expressing peritumoral fibroblasts show increased phosphorylation of SMAD family member 2 (SMAD2) and SMAD family member 3 (SMAD3) [41], suggesting the primary role of TGFβ-SMAD signaling in the induction of myCAF phenotypes. Intriguingly, recombinant TGFB1 also downregulated interleukin 1 receptor in PSCs, leading to a decreased induction of iCAF features [41]. These lines of evidence suggest the key role of TGFβ signaling in regulating myCAF and iCAF phenotypes.

The activation of hedgehog signaling by the tumor cell-derived Sonic Hedgehog and Indian Hedgehog also plays a key role in balancing fibroblast subpopulations and collagen in various diseases, including PDACs [133]. The pharmacological inhibition of the Hedgehog cellular signaling pathway in murine PDACs reduced collagen I deposition with a reduced proliferation in ACTA2-positive myofibroblasts, as well as an increased proliferation of ACTA2-negative cells [134]. In this GEMM model, short-term inhibition of the Hedgehog signaling pathway increased the delivery and effect of gemcitabine, resulting in the transient stabilization of pancreatic cancers [134]. However, a phase II clinical trial testing the benefit of the Hedgehog pathway blockade in PDACs paradoxically showed a poorer outcome in patients with PDAC when combined with gemcitabine compared to gemcitabine alone [135]. This result is consistent with previous findings showing that the genetic or long-term pharmacologic inhibition of Hedgehog signaling pathways in murine PDAC models suppressed desmoplastic stroma but accelerated tumor progression and pancreatic tumorigenesis [136,137,138]. Therefore, the Hedgehog signaling pathway generally expands myofibroblast-associated, collagen-rich stroma, contributing to the latent suppression of PDAC progression.

Desmoplastic stroma likely plays an important role in regulating anti-tumor immunity in the pancreatic cancer microenvironment. Various aforementioned stroma-reducing approaches have resulted in increased tumor-infiltrating immune cells in PDAC tissues, highlighting the importance of desmoplastic stroma as a physical and biological barrier to immune surveillance. To date, most PDACs have been refractory to immunotherapies. One of the major reasons for this is the immunosuppressive milieu of the desmoplastic stroma [139,140,141]. Indeed, human sample-based evidence indicates that the mean density of CD8+ T cells in the tumor center is less than half in the tumor margin [126]. Anti-tumor immune indicators, including neoantigens, T cell densities, and tertiary lymphoid structures, have been associated with prolonged survival in patients with PDACs [44,126,142]. Therefore, stroma-reducing approaches may contribute to unleashing the power of the latent anti-tumor immunity of PDAC and, theoretically, may enhance T cell-mediated immunotherapies. One such approach is targeting FAP-expressing CAFs, which have been associated with local immunosuppression in solid tumors [91,143,144,145]. A recent single-cell analysis has identified specific CAF subpopulations within FAP-expressing CAFs that are associated with immunotherapy resistance [146]. Previous studies have also suggested that FAP-dominant fibroblasts likely contribute to the spatial exclusion of CD8 T cells within PDAC tumor beds, regardless of baseline levels of CD8+ cell infiltration [44]. Therefore, FAP-expressing CAFs could be an attractive target for stromal therapies in solid organs and several FAP-targeting agents have been appreciated in clinical trials [135]. In pancreatic cancer, the CXCL12 chemokine derived from FAP-expressing CAFs has gained recognition as an attractive therapeutic target [147]. In the pancreatic cancer microenvironment, FAP-expressing CAFs have been a major source of CXCL12, which can bind to KRT19 (cytokeratin 19) at the surface of pancreatic cancer cells [148,149]. In murine PDAC models, CXCL12 inhibition has promoted intratumoral T cell aggregation and synergized with the PDCD1 pathway blockade [148]. Moreover, dual blockade of the C-X-C motif chemokine receptor 4 (CXCR4)–CXCL12 axis and the PDCD1 immune checkpoint, with or without chemotherapy, has shown encouraging results in the treatment of metastatic PDACs in phase II clinical trials [64,65].

### 4.2. Locoregional Levels

Tissue-based image analyses of human PDACs have visualized a wide range of locoregional heterogeneity in the proportions of fibroblast subpopulations and collagen, even in a patient (Figure 1) [44]. In addition, stroma types defined by these fibroblast subpopulations and collagen correlated with tumor morphologies and immune phenotypes [44]. For example, tumor cells form well-defined glands within the collagen-rich, fibroblast-poor stroma [44]. In contrast, cancer cells have a poorly differentiated morphology in the fibroblast-rich stroma, especially when enriched by FAP dominant fibroblasts [44]. A recent study has also indicated that the human PDAC microenvironment is composed of “sub-TMEs”, histologically definable regional TME states characterized by fibroblast plasticity, which shapes regional immunity and epithelial phenotypes and influences the clinical metrics of tumor aggressiveness [150]. Moreover, the tumor–normal interface appears to provide an additional complexity to the intratumoral PDAC heterogeneity by serving as a considerably different tumor−immune microenvironment from the tumor center [44,126].

### 4.3. Organ Levels

Pancreatic cancers frequently metastasize to distant organs, including the liver and lung. Unlike primary tumors, most metastatic PDACs in the liver and lung have not developed a dense desmoplastic stroma. Histological analyses have shown that metastatic tumors from PDACs often show a replacement growth pattern without a significant desmoplastic reaction [151]. Pulmonary metastatic tumors from PDACs frequently show a lepidic growth-like proliferation with a paucity of desmoplastic stroma [152] and can mimic primary lung adenocarcinomas. However, several studies have attested to the importance of stromal factors in regulating metastatic diseases. For example, Lenk et al. have demonstrated that the hepatic stromal microenvironment is essential for dictating tumor cell dormancy in liver metastases of PDACs [153]. In addition, immune-regulatory pathways in the tumor microenvironment of metastatic PDACs appear to differ in the liver and lung [127]. On the other hand, pancreatic cancers can easily disseminate into the abdominal cavity to develop peritoneal implants with a similar desmoplastic stroma to primary tumors. Importantly, metastatic tumors in the liver and lung have been monoclonal, while peritoneal implants have frequently been polyclonal [154]. These lines of evidence suggest the presence of organ-specific stromal characteristics in metastatic PDACs. Since metastasis has been a major cause of cancer-specific death in many PDAC patients, a deeper understanding of organ-level heterogeneity is needed to help develop meaningful interventions in the future.

## 5. Intertumoral Stromal Heterogeneity and Clinical Implications

Different CAF subpopulations appear to exert tumor-promoting and tumor-restricting functions in PDACs [106,155,156,157,158,159], indicating the presence of so-called “tumor-promoting CAFs” and “tumor-restricting CAFs”. Indeed, preclinical and population-based studies focusing on simple CAF markers, including ACTA2, have obtained conflicting results in the prognostic significance of CAFs in PDACs, although many studies suggested inverse associations between CAF amounts and patient survival time [159,160,161,162,163,164,165,166,167,168]. In murine PDAC models, ACTA2 deletion has developed an undifferentiated tumor and promoted tumor progression [169]. Depleting FAP-expressing CAFs results in the prolonged survival of murine PDAC models, whereas the depletion of ACTA2-expressing CAFs leads to shortened survival [170]. Moreover, this study has shown improved gemcitabine efficacy as well as synergy with the PDCD1 pathway blockade when IL6 is selectively ablated in ACTA2-expressing CAFs [170]. Tumor-promoting CAFs and tumor-restricting CAFs are likely mixed, even in a single clinical tumor, and this balance within a PDAC could be modified by several CAF-targeted approaches (i.e., Hedgehog inhibitors and anti-FAP agents), which may lead to altered clinical outcomes. Indeed, while the ablation of ACTA2-expressing CAFs likely does not influence the number of FAP-expressing stromal cells [169], the depletion of FAP-expressing CAFs has concurrently decreased the number of ACTA2-expressing CAFs [106]. Therefore, a deeper understanding of the landscape of intertumoral heterogeneity in tumor-promoting and tumor-restricting CAFs is needed to develop precision stromal therapies against human PDACs.

There is accumulating evidence of a wide range of intertumoral heterogeneity in stromal activity among PDAC populations. On-tissue quantitative analyses of 215 surgically resected PDACs have shown that the mean occupancy rate of collagen in tumor tissues was 38.4%, while that of fibroblastic cell populations expressing ACTA2 and/or FAP was 33.3% (unpublished data related to our previous analyses [44]). However, the compositions of these desmoplastic stromal elements have considerably differed between tumors and show distinct prognostic significance in patients with PDACs (Figure 1). In accordance with this finding, the ratios of ACTA2-expressing areas to collagen areas in whole-tissue sections of PDAC are linked to shortened survival [171]. Similarly, Mahajan et al. have shown that fibrolytic PDAC stroma (high ACTA2/low collagen expression) showed the shortest progression-free survival, while ACTA2 or collagen expression alone did not show any correlation with PDAC outcomes [168].

On the other hand, the virtual microdissection of stroma-derived gene expressions in human PDAC specimens has defined two transcriptome stroma subtypes, “activated stroma” and “normal stroma”, as associated with clinical outcomes [172]. The “normal stroma” transcriptome subtype has been associated with better PDAC outcomes [172] and is tightly linked to the tissue-based, collagen-rich, fibroblast-poor stroma (Figure 1) [44]. Chen et al. also demonstrated that the selective deletion of type I collagen in ACTA2-expressing myofibroblasts in murine PDAC augmented immunosuppression and promoted tumor progression [173]. Bhattacharjee et al. have shown that mechanical restriction by collagen opposes the tumor-promoting effects of hepatic stellate cell-derived CAFs in metastatic liver models [174]. These lines of evidence attest to the importance of the imbalance between the amounts of collagen and CAFs in the direction of the overall clinical behavior of human PDACs.

## 6. Future Direction of Targeting Desmoplastic Stroma of Pancreatic Cancer

The desmoplastic stroma is an essential part of pancreatic carcinogenesis and could be one of the modifiable vulnerabilities when treating PDAC. Multiple clinical trials focusing on the depletion of PDAC-associated desmoplastic stroma have been undertaken, but most have failed. This review introduced several successful and unsuccessful examples of stroma-modifying approaches against PDACs. These prior studies have highlighted caveats to keep in mind in the clinical application of stroma-targeting agents against PDAC. Firstly, the use of any stromal therapy alone appears to be ineffective for PDACs, but several anti-stromal agents can be significantly beneficial when used with several cytotoxic drugs, including standardized chemotherapy regimens and immunotherapies. Anti-stromal therapies likely break down stroma-dependent machinery (i.e., tumor cell transdifferentiation), which may lead to a reduction in intratumoral heterogeneity and reinitiation of tumor-cell-intrinsic treatment sensitivity. Therefore, it would be warranted to determine an optimal set of stroma-modifying agents and anti-tumor cell drugs to develop a novel, combined stromal therapy against PDACs. Secondly, caution should be taken regarding the detrimental effects of stroma-modifying drugs. For example, a high dose of stroma-modifying treatment (i.e., hyaluronan-degrading PEGPH20) may have an anti-tumor effect but can frequently be harmful in PDAC patients and even in healthy individuals. As stroma-modifying agents may not be a hub player, but rather an enhancer in combined stromal therapies, we should find a minimum required dose for each combined stromal therapy. Lastly, because recent findings have attested to the importance of intertumoral stromal heterogeneity associated with clinical impact in patients with PADC [44,150,168,172], appropriate patient selection may be crucial for the success of stroma-targeted therapies, as with many clinically established targeted treatments. To that end, future studies are needed to develop biomarkers that could predict the clinical benefit of each combined stromal therapy against PDACs.

## 7. Conclusions

Studies on the multilayered levels of stromal heterogeneity have likely identified promising stromal targets in pancreatic cancers and have shown evidence that several combined stromal therapies could become “game-changers” in fighting this hard-to-treat cancer.

**Use of Standardized Official Symbols**

We use HUGO (Human Genome Organization)-approved official symbols for genes and gene products, including ACTA2, BRCA1, BRCA2, CDKN2A, CD274, CD44, CD8, CTLA4, CXCL12, CXCR4, DES, FAP, GFAP, GLI1, HOXB6, IL1A, IL6, ITGA6, ITGB4, KRAS, KRT19, LAMA3, LAMB3, LAMC2, LIF, LOX, LOXL2, NTRK, PDCD1, PDGFR, PDPN, RELA, SMAD2, SMAD3, SMAD4, STAT3, S100A4, TGFB1, and TP53, all of which are described at www.genenames.org. Gene names are italicized and all upper-case when referring to human genes and gene names are italicized with the first letter upper-case and the remaining lower-case for mouse genes. Protein names are not italicized with all upper-case letters, regardless of the organism.

## Figures and Tables

**Table 1:** Selected therapeutic stromal targets and clinical trials.

Target	Candidate Drug and Combination Regimen	Drug Type	Mechanism	Outcomes
LOXL2	Simtuzumab plus gemcitabine	Blocking antibody	Destabilizes collagen networks	Negative outcome (phase II [58])
Renin−angiotensin system	Losartan plus FOLFIRINOX followed by chemoradiotherapy (as neoadjuvant therapy)	Small molecule inhibitor	Reduces collagen and hyaluronan	Downstaging (phase II [59])
Focal adhesion kinases	Defactinib plus pembrolizumab and gemcitabine	Small molecule inhibitor	Prevents integrin signaling	Clinical trials in phase I completed ([60], NCT02546531)
Hyaluronan	PEGPH20 plus nab-paclitaxel and gemcitabine	Enzyme	Degrades hyaluronan	Improved PFS (phase II [61])
	PEGPH20 plus modified FOLFIRINOX			Detrimental effects (phase Ib/II [62])
	PEGPH20 plus nab-paclitaxel and gemcitabine			Negative outcome (phase III [63])
Hedgehog	Saridegib plus gemcitabine	Small molecule inhibitor	Prevents/reduces CAF activation	Worse clinical outcome (phase Ib/II, NCT01130142)
CXCR4-CXCL12	Motixafortide plus pembrolizumab and chemotherapy	Small molecule inhibitor	Interferes with CAF signaling	Improved objective response rate (phase II [64,65])
FAP	RO6874281	Small molecule inhibitor	Interferes with CAF function	Clinical trials in phase I ongoing (NCT02627274)
